# Mining the interpretable prognostic features from pathological image of intrahepatic cholangiocarcinoma using multi-modal deep learning

**DOI:** 10.1186/s12916-024-03482-0

**Published:** 2024-07-08

**Authors:** Guang-Yu Ding, Wei-Min Tan, You-Pei Lin, Yu Ling, Wen Huang, Shu Zhang, Jie-Yi Shi, Rong-Kui Luo, Yuan Ji, Xiao-Ying Wang, Jian Zhou, Jia Fan, Mu-Yan Cai, Bo Yan, Qiang Gao

**Affiliations:** 1 Department of Liver Surgery and Transplantation, Liver Cancer Institute, Zhongshan Hospital, and Key Laboratory of Carcinogenesis and Cancer Invasion of Ministry of Education, Fudan University, No.180, Feng Lin Road, Shanghai, 200032 China; 2 School of Computer Science, Shanghai Key Laboratory of Intelligent Information Processing, Fudan University, No.2005, Song Hu Road, Shanghai, 200433 China; 3 Department of Pathology, Zhongshan Hospital, Fudan University, Shanghai, 200032 China; 4 Institute of Biomedical Sciences, Fudan University, Shanghai, 200032 China; 5 State Key Laboratory of Oncology in South China, Collaborative Innovation Center for Cancer Medicine, Sun Yat-Sen University Cancer Center, No.651 Dongfeng Road East, Guangzhou, 510060 China; 6 State Key Laboratory of Genetic Engineering, Fudan University, Shanghai, 200433 China

**Keywords:** Cholangiocarcinoma, Deep learning, Interpretable model, Prognosis, Tumor morphology

## Abstract

**Background:**

The advances in deep learning-based pathological image analysis have invoked tremendous insights into cancer prognostication. Still, lack of interpretability remains a significant barrier to clinical application.

**Methods:**

We established an integrative prognostic neural network for intrahepatic cholangiocarcinoma (iCCA), towards a comprehensive evaluation of both architectural and fine-grained information from whole-slide images. Then, leveraging on multi-modal data, we conducted extensive interrogative approaches to the models, to extract and visualize the morphological features that most correlated with clinical outcome and underlying molecular alterations.

**Results:**

The models were developed and optimized on 373 iCCA patients from our center and demonstrated consistent accuracy and robustness on both internal (*n* = 213) and external (*n* = 168) cohorts. The occlusion sensitivity map revealed that the distribution of tertiary lymphoid structures, the geometric traits of the invasive margin, the relative composition of tumor parenchyma and stroma, the extent of necrosis, the presence of the disseminated foci, and the tumor-adjacent micro-vessels were the determining architectural features that impacted on prognosis. Quantifiable morphological vector extracted by CellProfiler demonstrated that tumor nuclei from high-risk patients exhibited significant larger size, more distorted shape, with less prominent nuclear envelope and textural contrast. The multi-omics data (*n* = 187) further revealed key molecular alterations left morphological imprints that could be attended by the network, including glycolysis, hypoxia, apical junction, mTORC1 signaling, and immune infiltration.

**Conclusions:**

We proposed an interpretable deep-learning framework to gain insights into the biological behavior of iCCA. Most of the significant morphological prognosticators perceived by the network are comprehensible to human minds.

**Graphical Abstract:**

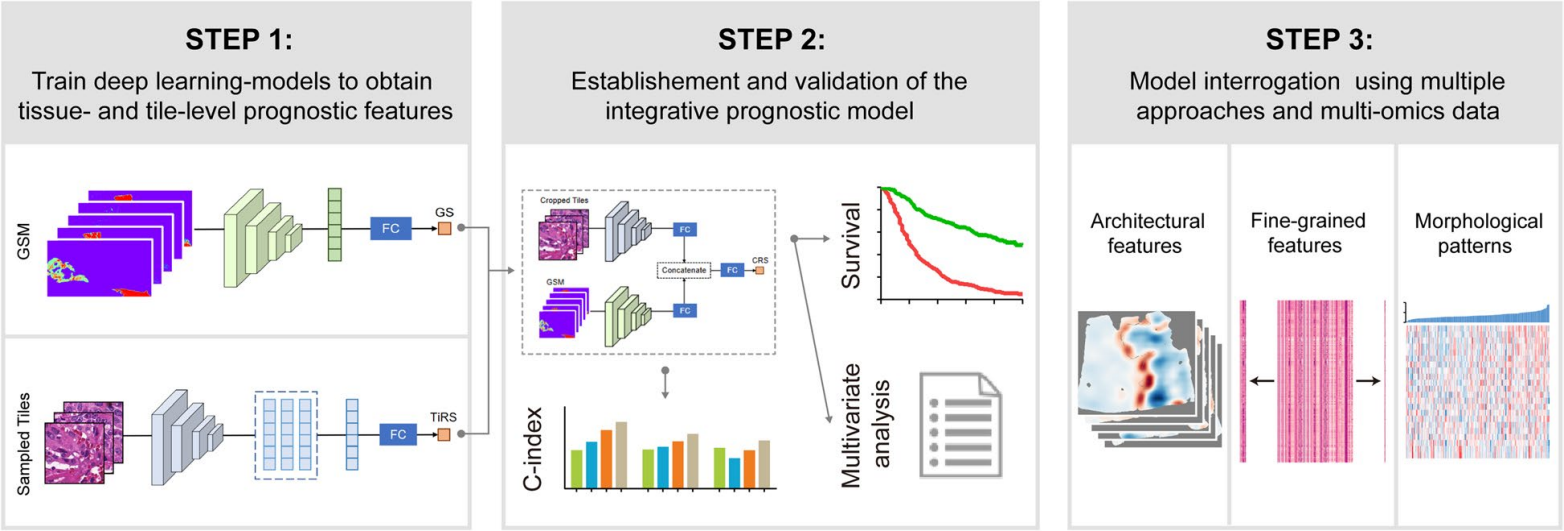

**Supplementary Information:**

The online version contains supplementary material available at 10.1186/s12916-024-03482-0.

## Background

Deep learning has invoked tremendous insights into cancer prognostication and treatment efficacy prediction based on whole-slide images (WSIs) [1–3]. The performance of deep learning-based prognostic model triumphed over almost all other conventional methods when applying to a wide range of malignancies, showing the enormous potential in using it for more personalized clinical care [4–6]. These pioneering studies also showed that deep learning approaches could extract essential pathological features that were morphological determinants of prognosis. However, despite the promising prospects, several major obstacles must be addressed before practical application.

Current limitations of deep learning approaches include the tendency of shortcut learning, poor generalizability, and limited interpretability [7], all derived from the “black box” nature of the neural network. Effective networks commonly used consist of extremely complex layers connected via many nonlinear intertwined relations, thus making it very difficult to comprehend the transformation from inputs to outputs. Therefore, “understanding the model” is a critical issue, in order to (i) exclude data artifacts and non-universal features to avoid shortcut learning, (ii) gain the required confidence in deep learning models’ outputs, and (iii) exploit key features and novel biological behavior of tumor that have been overlooked [1]. In principle, there are two main strategies for model interpretation: model-based explanation and post hoc explanation [8]. The model-based one refers to models with predetermined confinements which mostly relied on prior knowledge of certain disease, while post hoc explanation refers to analyzing a trained model to achieve insight into learned relationships. The combination of the two strategies should be complementary to each other and might benefit the decipherment of the black box. With this in mind, we designated intrahepatic cholangiocarcinoma (iCCA) to establish a practical deep learning model for prognostication and in-depth interpretation.

iCCA is a desmoplastic cancer with complex tissue composition, varied prognosis among patients, and distinct molecular background, making it ideal for deep learning modeling [9–11]. The morphological information hidden in pathological sections of iCCA reflects the overall effect of the microenvironment on the behavior of tumor cells. Herein, using pathological images of 4 independent iCCA cohorts from 2 cancer centers, we developed and validated a stepwise prognostic model for this malignancy with excellent accuracy, generalizability, and robustness. Next, the established model was intensively interrogated by multiple approaches, generating a human-interpretable feature library of unprecedented resolution and detail. Lastly, we explored the footprints of molecular alterations in morphological changes leveraging the multi-omics data of the studied cohort.

## Methods

### Study populations and pathological sections

Four independent iCCA cohorts were included in this study (cohorts T, V1, V2, and FU-iCCA) with a total of 941 patients [12, 13]. Cohorts T and V1 totally comprised 586 iCCA patients who underwent surgical resection between 2004 and 2015 at Zhongshan Hospital of Fudan University. Cohort T (as the training cohort) comprised 373 iCCA patients, and the remaining 213 patients were included in cohort V1 as the internal validation. Cohort V2 (as the external validation cohort) comprised 168 iCCA patients from the Sun Yat-sen University Cancer Center resected between 1999 and 2013. The FU-iCCA cohort was established previously by us with multi-omics data which included 187 patients resected between 2014 and 2018 at Zhongshan Hospital [13]. All patients had pathologically proven iCCAs and underwent curative resection. Patients with hilar or extrahepatic cholangiocarcinoma and a mixed type of primary liver cancer were excluded. None of the patients received any molecular targeted or immune therapy before surgery or during the follow-up period. The study protocol was conducted in accordance with ethical guidelines (Declaration of Helsinki and Istanbul) and approved by the Institutional Ethics Committee of Zhongshan Hospital. Written informed consent was obtained from all subjects prior to participating in the study for the use of surgical specimens and related clinical data.

The pathological H&E sections of each patient were scanned for WSIs. In total, 1782 WSIs from all four cohorts were collected. After initial screening, 321 were excluded due to poor quality (including contamination, depigmentation, and overlapping), and the remaining were 673 WSIs of 373 patients in cohort T, 433 WSIs of 213 patients in cohort V1, 168 WSIs of 168 patients in cohort V2, and 187 WSIs of 187 patients in the FU-iCCA cohort (multiple WSIs were available for some cases in cohorts T and V1). Detailed information is shown in Table 1 and Additional file 1: Supplementary methods, and the study population and overall frameworks of the neural networks used in this study are shown in Additional file 2: Fig. S1.

**Table 1:** Demographics and clinicopathologic characteristics of patients with iCCA

Characteristic	Cohort T	Cohort V1	Cohort V2	FU-iCCA	*P* value	*P* value	*P* value
	**(*n* = 373)**	**(*n* = 213)**	**(*n* = 168)**	**(*n* = 187)**	**T vs V1**	**T vs V2**	**T vs FU-iCCA**
Age, years							
Mean (SD)	60.3 (9.9)	59.9 (11.2)	53.1 (12.6)	61.2 (10.7)	0.821*	< 0.001*	0.232*
Range	(28.0–85.0)	(27.0–88.0)	(20.0–86.0)	(28.0–86.0)			
Sex							
Male (%)	222 (59.5%)	122 (57.3%)	113 (67.3%)	108 (57.8%)	0.596	0.079	0.616
Female (%)	151 (40.5%)	91 (42.7%)	55 (32.7%)	79 (42.2%)			
History of hepatitis							
Yes (%)	151 (40.5%)	82 (38.5%)	91 (54.2%)	49 (26.2%)	0.637	0.003	0.001
No (%)	222 (59.5%)	131 (61.5%)	77 (45.8%)	136 (72.7%)			
History of hepatolithiasis							
Yes (%)	66 (17.7%)	42 (19.7%)	18 (10.7%)	12 (6.4%)	0.543	0.027	< 0.001
No (%)	307 (82.3%)	171 (80.3%)	150 (89.3%)	175 (93.6%)			
History of parasite infection							
Yes (%)	21 (5.6%)	14 (6.6%)	5 (3.0%)	7 (3.7%)	0.643	0.101	0.347
No (%)	352 (94.4%)	199 (93.4%)	163 (97.0%)	180 (96.3%)			
HBsAg							
Positive (%)	116 (31.1%)	61 (28.6%)	88 (52.3%)	45 (24.1%)	0.533	< 0.001	0.261
Negative (%)	257 (68.9%)	152 (71.4%)	79 (47.7%)	142 (75.9%)			
Anti-HCV							
Positive (%)	8 (2.1%)	6 (2.8%)	1 (0.6%)	0 (0.0%)	0.608	0.114^†^	0.056^†^
Negative (%)	365 (97.9%)	207 (97.2%)	167 (99.4%)	187 (100.0%)			
γ-GT, U/L							
Mean (SD)	120.1 (172.6)	115.9 (166.9)	137.2 (242.5)	118.9 (149.3)	0.627*	0.046*	0.886*
Range	(14.0–1753.0)	(12.0–1337.0)	(16.7–1945.4)	(2.0–1003.0)			
CA19-9, U/ml							
Mean (SD)	898.7 (2358.7)	1039.1 (2550.7)	653.3 (2128.8)	788.1 (2120.9)	0.503*	0.787*	0.368*
Range	(0.0–10000.0)	(0.0–10000.0)	(0.6–19,671.0)	(0.6–10,000)			
Size, cm							
Mean (SD)	6.2 (2.9)	6.1 (2.8)	6.0 (2.9)	6.3 (2.6)	0.748*	0.520*	0.441*
Range	(1.0–20.0)	(1.0–15.0)	(1.0–17.0)	(1.0–15.0)			
Tumor number							
Single (%)	320 (85.8%)	175 (82.2%)	146 (86.9%)	147 (78.6%)	0.243	0.482	0.034
Multiple (%)	53 (14.2%)	38 (17.8%)	22 (13.1%)	40 (21.4%)			
Liver background							
Cirrhosis (%)	65 (17.4%)	36 (16.9%)	20 (12.0%)	19 (10.2%)	0.871	0.114	0.026
Fibrosis (%)	64 (17.2%)	33 (15.5%)	49 (29.2%)	67 (35.8%)	0.602	0.020	< 0.001
Steatosis (%)	21 (5.6%)	7 (3.3%)	18 (10.8%)	38 (20.3%)	0.201	0.031	< 0.001
Cholestasis (%)	30 (8.0%)	10 (4.7%)	20 (12.0%)	13 (6.9%)	0.122	0.001	0.220
AJCC/UICC 8th TNM stage							
I (%)	249 (66.8%)	134 (62.9%)	79 (47.0%)	50 (26.7%)	0.336	< 0.001	< 0.001
II (%)	49 (13.1%)	40 (18.8%)	27 (16.1%)	63 (33.7%)			
III (%)	59 (15.8%)	31 (14.6%)	33 (19.6%)	66 (35.3%)			
IV(%)	16 (4.3%)	8 (3.8%)	29 (17.3%)	8 (4.3%)			
Lymph node metastasis							
Yes (%)	68 (18.3%)	33 (15.9%)	37 (22.0%)	42 (22.5%)	0.454	0.363	0.223
No (%)	303 (81.7%)	175 (84.1%)	131 (78.0%)	145 (77.5%)			
Subtype for iCCA							
Small duct type (%)	183 (49.7%)	102 (48.3%)	118 (70.2%)	109 (58.3%)	0.748	< 0.001	0.049
Large duct type (%)	185 (50.3%)	109 (51.7%)	50 (29.8%)	78 (41.7%)			
Differentiation							
Well (%)	24 (6.4%)	23 (10.8%)	18 (10.7%)	1 (0.1%)	0.038	0.113	< 0.001
Moderate (%)	234 (62.7%)	113 (53.1%)	91 (54.2%)	147 (78.6%)			
Poor (%)	115 (30.8%)	77 (36.2%)	59 (35.1%)	39 (20.9%)			
Microvascular invasion							
Yes (%)	60 (16.5%)	44 (21.5%)	50 (30.1%)	87 (46.5%)	0.144	0.021	< 0.001
No (%)	303 (83.5%)	161 (78.5%)	116 (69.9%)	100 (53.5%)			
Macrovascular invasion							
Yes (%)	26 (7.0%)	16 (7.5%)	18 (10.7%)	3 (1.6%)	0.807	0.196	0.008
No (%)	347 (93.0%)	197 (92.5%)	150 (89.3%)	184 (98.4%)			

*Chi-square test was used for other tests*

*Abbreviations*: *iCCA*, intrahepatic cholangiocarcinoma; *HBsAg*, hepatitis B surface antigen; *HCV*, hepatitis C virus; *γ-GT*, γ-glutamyltransferase

^*^Mann–Whitney *U* test

^†^Fisher’s exact test

### Classification networks and global segmentation map

Raw image data were preprocessed to remove meaningless background (Otsu method) [6]. To classify and segment distinct anatomical subregions, 89 WSIs of 74 patients from cohort T were randomly selected for manual annotation. First, principle anatomical subregions, such as tumor tissue (TT), peri-tumor liver tissue (LT), hemorrhage and necrosis region (HN), and tertiary lymphoid structures (TLSs), were directly delineated by senior pathologists using QuPath [14]. Subsequently, these images were divided into non-overlapping small patches (256 × 256 pixels), referred to as labeled tiles. Given the difficulty of directly delineating the demarcation between tumor parenchyma and stroma, we alternatively dichotomized tiles derived from tumor tissues into parenchyma (TT-p) and stroma (TT-s). These labeled tiles established the ground-truth for the classification networks. The detailed framework for the classification networks could be found in Additional file 1: Supplementary methods. Then, the global segmentation map (GSM) activated by class could be generated for each WSI.

### Prognostic models

We established and compared several prognostic models according to inputs from different dimensions. For prognostic model 1, we took each patient as a sample, with the survival time as the label and GSM as the input. After training, each patient’s risk score could be generated by this model, as a relative value to assess prognosis. For prognostic model 2, we also took each patient as an example, but the input was changed to tiles sampled within the WSIs. The tiles for prognostic model 2 were 256 × 256 pixels in size. The sampling strategies included random sampling and category-based sampling, and the effect of different tile counts and magnification scales on network performance was also tested. The optimal and most efficient sampling strategy (sampling for 32 tile counts at 4 × magnification scale in the region of tumor parenchyma) was then determined in the final model, and the risk score for each patient could be generated. For the integrated prognostic model, both GSM and sampled tiles were utilized as inputs to the network, and a consensus risk score could be generated for each patient.

In cohorts T and V1, multiple WSIs were available for some cases, and inter-section discrepancies of risk scores were observed. For multiple WSIs, the minimal, maximal, and mean risk scores were compared for their predictive accuracy, and the variation of risk scores within one case was also evaluated in the form of standard deviation. Details of the prognostic networks could be found in Additional file 1: Supplementary methods.

### Identification of prognosis-related architectural features

To unveil the “black box” of the prognostic model, we first applied the occlusion sensitivity map (OSM) [15] to visualize the prognosis-related features of GSM. By calculating the resulting difference in risk score using GSM occlusion, we generated coarse sensitivity heatmaps where different colors indicated higher and lower risks of death. Detailed methodology was provided in Additional file 1: Supplementary methods.

Second, we deconstructed the GSM by extracting predefined architectural parameters. These architectural parameters included area ratios of HN/TLS/TT-p/TT-s to TT/LT, distribution variance of TLS and HN, smoothness of invasive margin, and distances between TLS and invasive margin. In implementation, area ratios were based on amounts of pixels, smoothness of invasive margin was calculated by Sobel operator, and distances were defined as minimum distances between centers of TLS clusters and invasive margin.

### Extraction of prognosis-related tile-level features

To interpret the prognosis-related tile-level features perceived by the network, we applied CellProfiler® to automatically extract quantified image vector from tiles of high risk and low risk. The workflow for image processing and vector extraction has been described previously [16], and we adjusted the pipeline parameters to better apply the scenarios of pathological sections. Briefly, after exclusion of low-quality images and correction for uneven illumination, tumor cell nuclei were identified and segmented for each tile using CellProfiler version 2.1.0. Then, the nuclear area shape, intensity, texture, and radial distribution were measured for each nucleus. In total, a feature vector of 732 measurements was extracted and normalized to describe the summarized morphology of tumor cell nuclei in a tile. Each feature vector contained nuclei information of the mean, median, and standard deviation of nucleus size, contour line length, orientation, ellipticity, texture entropy, central moment, etc. Dimensionality reduction was conducted using truncated SVD [17]. The detailed methodology and CellProfiler pipeline used to process the images were provided in the Additional file 1: Supplementary methods.

### WES, RNA-seq, and proteomic analysis

WES, RNA-seq, and proteomic data were downloaded from Dong et al. [13]. The estimation for immune subgroups was performed using the method described by Danaher et al. based on RNA-Seq data [18]. To compute the scores of 50 hallmarks from MSigDB (http://www.gsea-msigdb.org/gsea/msigdb), we used GVSA in R package with parameter: method = “ssgsea”, kcdf = “Gaussian”, min.sz = 1, max.sz = 500.

### Statistical analysis

Statistical tests were performed using the SPSS (version 20.0; IBM, Armonk, NY, USA) and R software. Categorical variables were compared using Fisher’s exact test when more than 20% of cells had expected frequencies less than 5; otherwise, the chi-square test was used. Continuous variables were compared using the Mann–Whitney *U* test. The performance of the prognostic model was assessed by Harrell’s concordance index (C-index) and compared with the rcorrp.cens package in Hmisc in R. Survival analysis was performed using Kaplan–Meier and compared with the log-rank test to estimate the survival probability according to risk scores. Multivariate Cox regression analyses were performed to identify independent variables associated with overall survival. Lasso regression model was used to identify image vectors associated with tile risk score. A two-sided *P* < 0.05 was considered statistically significant.

## Results

### Classification networks precisely distinguish anatomical subregions

**Fig. 1:**
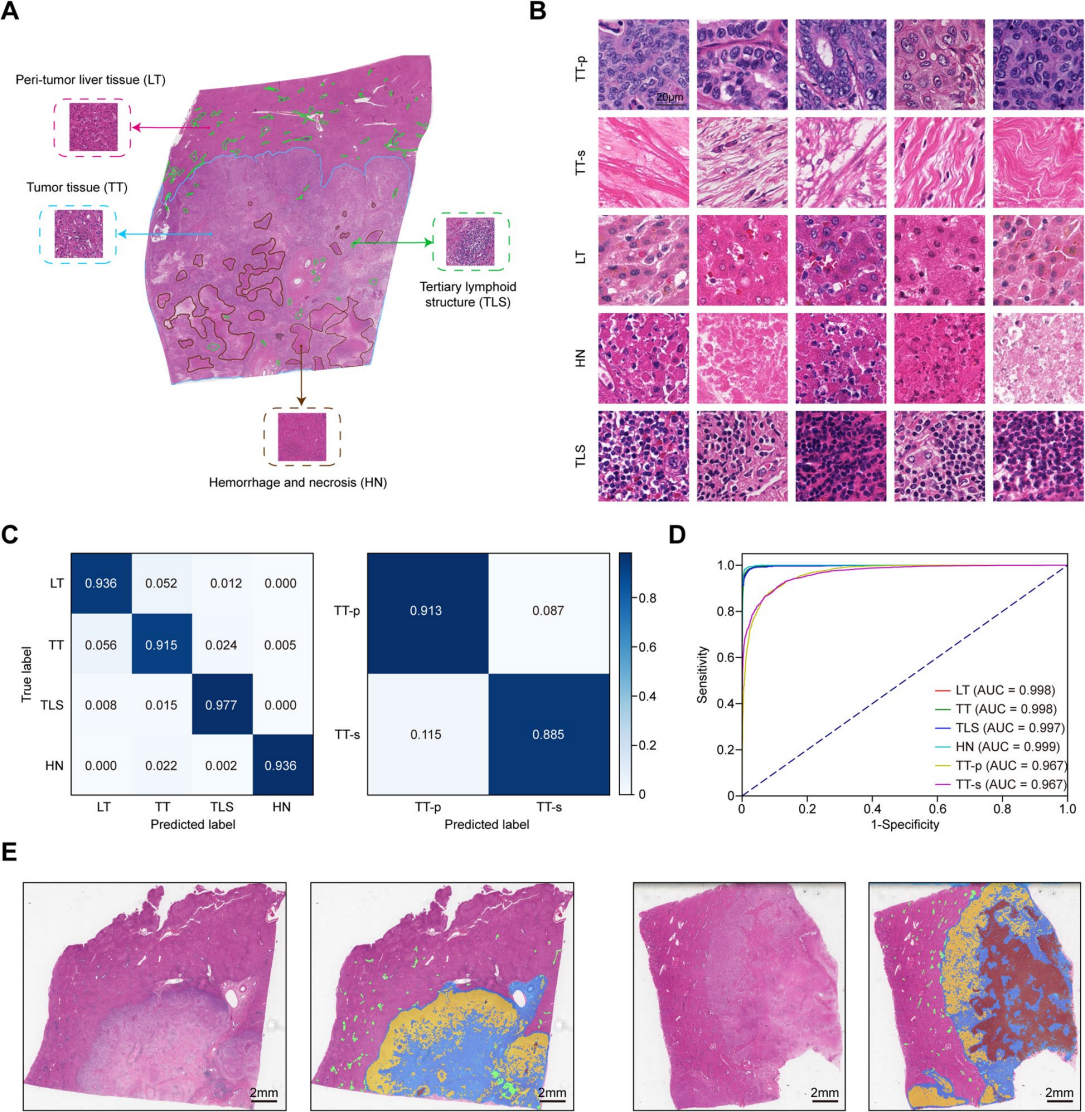
Classification networks and global segmentation map. **A** Representation of annotated WSIs. **B** Representation of labeled tiles for training the classification networks. **C** Normalized confusion matrices of the classification results. **D** Area under the curve (AUC) of each tissue category of the classification networks. **E** Two examples of the global segmentation map (GSM) activated by class for WSI. TT-p was masked in yellow, TT-s was masked in blue, HN was masked in brown, and TLS was highlighted in green. WSI, whole slide image; LT, peri-tumor liver tissue; TT, tumor tissue; TT-p, tumor parenchyma; TT-s, tumor stroma; TLS, tertiary lymphoid structure; HN, hemorrhage and necrosis

Generally, pathological sections of iCCA comprised 4 major anatomical subregions, including TT, LT, HN, and TLSs, which could be manually outlined relatively easy. Two experienced experts delineated the contours of the four types of tissue on 89 WSIs and checked by another pathologist independently (Fig. 1A). These WSIs were divided into tiles as mentioned in the methods (Fig. 1B). In total, 1,059,923 annotated tiles were sampled for training the classification networks. For 32,762 tiles annotated as TT, we further labeled them into parenchyma (TT-p) and stroma (TT-s) for subclassification, considering that iCCA is a highly desmoplastic cancer. After full training, the remaining 6552 labeled tiles were applied to test the performance of the classification networks using confusion matrices and area under the curve (AUC), all of which revealed excellent accuracy and distinguishability (Fig. 1C, D).

Next, we applied the classification networks to other WSIs in cohorts T, V1, and V2, and typical GSMs were shown in Fig. 1E. To further validate the authenticity of the outputs, we randomly selected 2000 tiles (400 tiles for each of the five regions) for each cohort (6000 tiles in total), and the classification results were checked by two experienced pathologists. The recognition accuracies of classification results were 0.986 in cohort T, 0.983 in cohort V1, and 0.983 in cohort V2, respectively. As shown in Additional file 3: Fig. S2, similar colors and contours were the main causes of misclassification. Overall, our classification networks performed well in distinguishing major subregions both internally and externally.

### Initial feature extraction from separate dimensions of pathological images

Based on prior knowledge, both architectural features (indicating the spatial organization of distinct anatomical subregions) and fine-grained features (indicating the morphology and texture of tumor cells) contain essential information related to patient outcomes [19]. To verify this knowledge and for better interpretability of the final model, we first established 2 preliminary prognostic models.

**Fig. 2:**
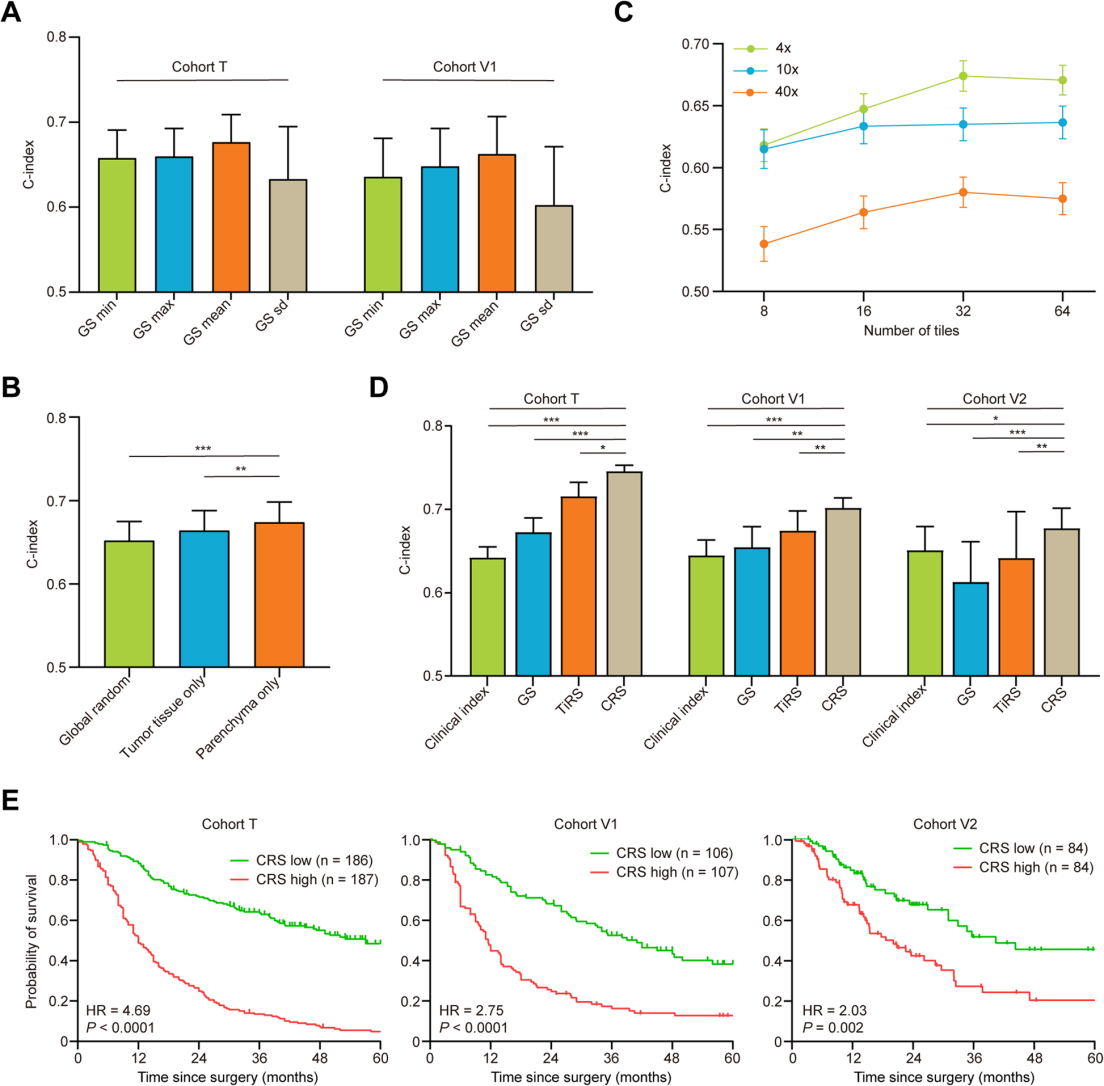
Evaluation of the predictive performance under various conditions. **A** C-indices via the minimal (GSmin), maximal (GSmax), mean (GSmean), and standard deviation (GSsd) of the GSs among patients with multiple WSIs in cohorts T and V1. **B** C-indices via TiRS according to different sampling methods. **C** C-indices via TiRS according to different tile counts under different magnification scales. **D** C-indices via GS, TiRS, CRS, and clinical index in cohorts T, V1, and V2. **E** Kaplan–Meier curves of survival for high and low CRS. The bars represent the 95% confidence intervals. *P < 0.05; **P < 0.01; ***P < 0.001; GS, GSM score; WSI, whole slide image; TiRS, tile risk score; CRS, consensus risk score; GSM, global segmentation map

For prognostic model 1, the global segmentation map was the input to train the network. The output of model 1 that we termed “GSM score (GS)” was a relative value to assess the prognostic risk of each WSI. For patients with multiple WSIs, the mean GS of all sections was adopted to be the representative risk score of this patient, based on the predictive performance (Fig. 2A). Overall, the C-indices via GS reached 0.672 for cohort T and validated by cohorts V1 and V2 without any tuning of the network (0.654 and 0.612 for cohorts V1 and V2, respectively).

For prognostic model 2, the inputs were sampled tiles, and the outputs were termed as “tile risk score (TiRS).” To saturate the network performance, sampling methods, tile counts, and magnification scales were iteratively optimized. We first compared the performances of the global random sampling method (irrespective of tissue category), tumor-tissue-only sampling method (irrespective of parenchyma or stroma), and tumor parenchyma-only sampling method, in which 32 tiles were sampled for each WSI as input. As shown in Fig. 2B, the performance of tumor parenchyma-only sampling method was significantly higher than that of the other 2 sampling methods (*P* < 0.05). This result endorsed the prior knowledge that prognosis-related fine-grained features were mostly derived from tumor cells [6]. Hence, we adopted the tumor parenchyma-only sampling method in the subsequent modeling. Next, we tested the effect of different magnification scales and tile counts on network performance. In respect of intra-tumor heterogeneity of iCCA, only with sufficient counts of tiles can the network comprehend the overall landscape of one tumor. As the tile counts increased, the C-index gradually increased until a platform appeared. Although increased magnification scale allows the network to capture more detailed features, the computing power required also increased exponentially. To achieve the maximal prognostication at the cost of minimal computing power, we determined 32 tile counts at 4 × magnification scale as the optimal combination (Fig. 2C). Similar with GS, for patients with multiple WSIs in cohorts T and V1, the mean TiRS was the better predictor of outcome (Additional file 4: Fig. S3). With the parameters determined, prognostic model 2 obtained a C-index of 0.715 in cohort T and validated by cohorts V1 and V2 without any re-training of the network (0.674 and 0.641 for cohorts V1 and V2, respectively).

Notably, GS and TiRS were independent of each other as prognosticators for iCCA (all *P* < 0.01 in cohorts T, V1, and V2), and the variations of GS and TiRS among multiple sections derived from one patient also significantly influenced prognosis (Fig. 2A and Additional file 4: Fig. S3A), showing the impact of intra-tumor heterogeneity in iCCA.

### The integrated prognostic model robustly predicts patient outcome

We have demonstrated that both GSM and sampled tiles were indispensable inputs for the prognostication of iCCA. In the integrated model, features from both dimensions were fed into the network synergistically, and the output was termed as “consensus risk score (CRS).” After optimization, the C-index of CRS reached 0.745 in the training cohort T, significantly better than either GS or TiRS (Fig. 2D, all *P* < 0.05). A major advantage of CRS was the generalizability proved by both internal and external validation cohorts. Without any modification or re-training, this model was directly applied in the remaining cohorts, and the C-indices retained 0.701 and 0.677 in cohorts V1 and V2, respectively. As expected, CRS was a superior prognostic predictor than Clinical index that derived from Cox model combing conventional clinicopathologic characteristics (Fig. 2D).

We further performed survival analysis using CRS by equally stratifying the patients into high- and low-risk groups. The survival curves showed that patients with high CRS showed significantly worse survival than those with low CRS (Fig. 2E). Multivariate analysis revealed that the prognostic power of CRS was independent of conventional clinicopathological characteristics (Additional file 5: Table S1), and CRS remained a predictor of survival in most clinicopathological subgroups, such as early or late TNM stages, small or large tumor size, and single or multiple tumors (Additional file 4: Fig. S3B).

### Deconstruction of prognostic model 1 reveals important architectural features

The direct combination of GS and TiRS (by using hazard linear combination) demonstrated slightly inferior yet comparable predictive performance with CRS (0.733 vs. 0.745 for cohort T, 0.695 vs. 0.701 for cohort V1, and 0.665 vs. 0.677 for cohort V2. Additional file 4: Fig. S3C) and therefore rationalized the stepwise interpretation of the final model by interrogating the preliminary models separately.

**Fig. 3:**
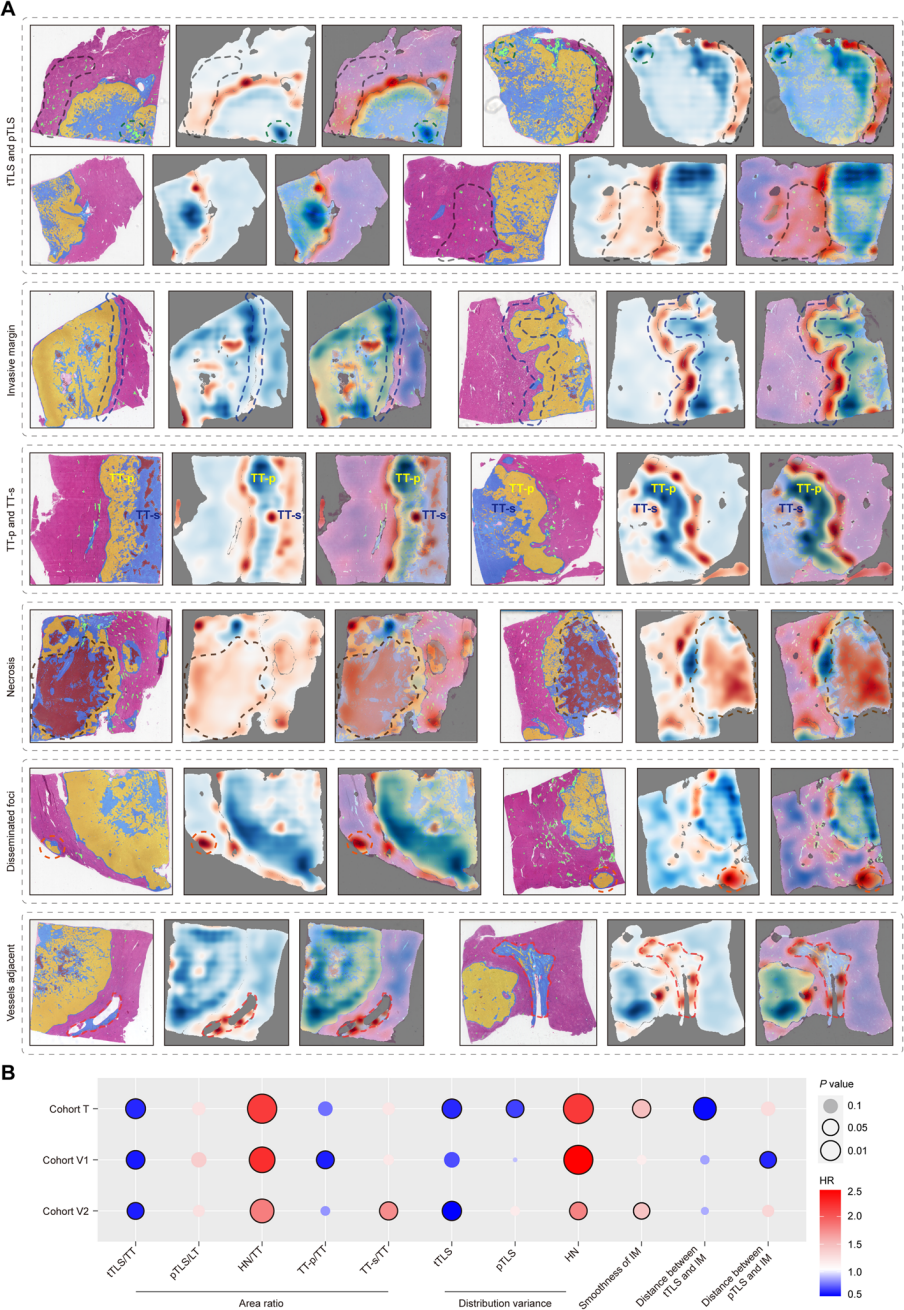
Deconstruction and visualization of architectural features. **A** Visualization of high-risk (masked in red) and low-risk (masked in blue) features attended by prognostic model 1 using occlusion sensitivity map (OSM). Each WSI was demonstrated in three forms: the original global segmentation map (left), the OSM (middle), and the merged image (right). The first panel showed the risk differences according to the presence and distribution of TLS (tTLS and pTLS were indicated by green circle and black circled curve respectively). The second panel highlighted the prognostic significance of the smoothness of invasive margin (blue circled curve). The third panel revealed the opposite prognostic impacts of TT-p (yellow) and TT-s (dark blue). The fourth, fifth, and sixth panels demonstrated other significant architectural features, including the presence of necrosis (brown circled curve), disseminated foci (orange circle), and adjacent micro-vessels (red circled curve). **B** The prognostic impacts of predefined architectural parameters in cohorts T, V1, and V2. Red circles indicate hazard ratios greater than 1, while blue circles indicate hazard ratios less than 1. The size of the circles indicates the *P* value. The area ratio of pTLS to LT, the area ratios of HN and TT-s to TT, the distribution variance of HN, and the smoothness of invasive margin showed potential associations with high risk, while the area ratios of tTLS and TT-p to TT and the distribution variance of tTLS were associated with low risk. WSI, whole slide image; TLS, tertiary lymphoid structure; tTLS, intra-tumor TLS; pTLS, peri-tumor TLS; TT, tumor tissue; LT, peri-tumor liver tissue; HN, hemorrhage and necrosis region; TT-p, tumor parenchyma; TT-s, tumor stroma

To interrogate prognostic model 1 explanations, we first used OSM to visualize prognostic relevance of different image regions. For visualization, the GSMs were masked in different colors, where redder and bluer indicated higher and lower risks of death. Several distinctive features were identified by our pathologists from these heatmaps (Fig. 3A). As shown in Fig. 3A, intra-tumor TLSs were specifically highlighted in dark blue, indicating a protective role against tumor. However, peri-tumor TLSs were masked in red, implying opposite prognostic impacts of TLSs located in and around the tumor [12]. The prognostic implications of invasive margin were determined by its smoothness or irregularity, with protrusions and depressions indicating the presence of tumor budding and suggesting high risks (Additional file 6: Fig. S4) [20]. Intriguingly, compared to tumor stroma which was notorious for its active tumor-promoting role [21] and frequently recognized as high-risk region by our network, tumor parenchyma was consistently linked with relatively low risk. Other risk-related features, including necrosis, disseminated foci, and micro-vessels adjacent to tumor, were all consistently displayed in red, which were comprehensible to human eyes [22, 23].

To quantify the findings above, we calculated several predefined architectural parameters, including the area ratios of different subregions, the distribution variance of TLS and HN, the smoothness of invasive margin, and the distances between TLS and invasive margin. Generally consistent with the observational findings, the area ratio of peri-tumor TLS to LT, the area ratios of HN and TT-s to TT, the distribution variance of HN, and the unsmoothness of invasive margin showed potential positive associations with dismal outcome, whereas the area ratios of intra-tumor TLS and TT-p to TT, and the distribution variance of intra-tumor TLS demonstrated positive correlations with favorable outcome (Fig. 3B). Nevertheless, these parameters did not fully represent the geographical complexity and topological patterns of distinct tumor regions and therefore could not fulfill the predictive power of the network (Additional file 7: Fig. S5).

Together, these findings proved the in-depth perception of essential prognosticators from GSM by prognostic model 1.

### Prognostic model 2 captures intrinsic morphological features from parenchyma tiles

**Fig. 4:**
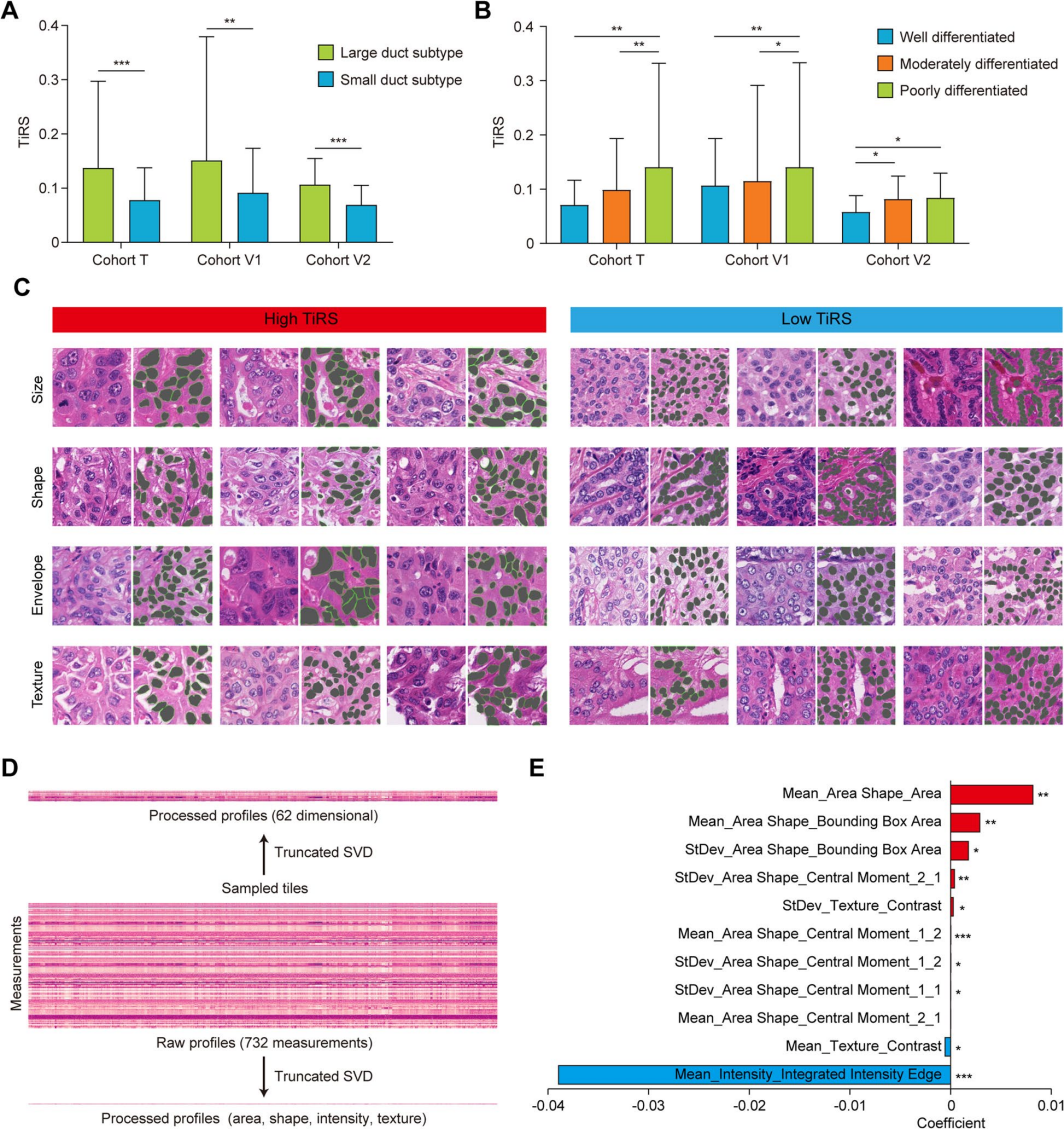
Quantified morphological analysis revealed fine-grained features. **A** Significant associations were found between TiRS and iCCA subtypes. **B** Poorly differentiated iCCAs had significantly higher TiRS than well/moderately differentiated tumors. The bars represent the 95% confidence intervals. **C** Representations of tiles from WSIs with high or low TiRS, tumor cell nuclei were automatically segmented by CellProfiler (masked in dark green and demonstrated on the right of each original tile). Tumor nuclei from high TiRS tiles exhibited larger size, more distorted shape, while their nuclear envelope and textural contrast were less prominent. **D** The raw profiles containing all 732 measurements of tumor cell nuclei were processed by dimensionality reduction. **E** The names and coefficients of the most significant measurements that correlated with TiRS after lasso regression. Red color indicated positive correlation and blue color indicated negative correlation. *P < 0.05; **P < 0.01; ***P < 0.001; TiRS, tile risk score; iCCA, intrahepatic cholangiocarcinoma; WSI, whole slide image

Prognostic model 2 focused on microscopic features, as determined by its inputs. We first tested if the model recognized pathologist-interpretable prognosticators such as iCCA subtype or tumor grade. According to the World Health Organization and European Network for the Study of Cholangiocarcinoma, iCCA can be classified into perihilar large duct subtype and peripheral small duct subtype, with significant differences in mucin production, the shape of tumor cells, and patient prognosis [24]. Compared to large duct subtype with inferior outcome, tiles from small duct subtype of iCCAs had significantly lower TiRS, which mutually corroborated with their favorable prognosis (Fig. 4A). The same association was also observed between differentiation grades and TiRS (Fig. 4B). These results reflected that prognostic model 2 had captured morphological features that defined conventional pathological characteristics.

Second, we focused on tumor cell nuclei and extracted quantifiable morphological vector using CellProfiler [16] to aid our understanding of the “black box.” To filter out potential features that were attended by the network, we randomly selected tiles from patients with high (top 20%) and low (bottom 20%) TiRS. In total, 2128 tiles were processed to segment tumor cell nuclei automatically by CellProfiler (Fig. 4C). The raw profiles containing all 732 measurements for each tile and the significant features emerged after dimensionality reduction were schematically showed in Fig. 4D. Area shape, intensity, and texture of the nuclei provided the most informative features that significantly associated with TiRS and prognosis. Lasso regression revealed that the most relevant measurements included the mean of nucleus size, the third-order central moment of nucleus shape, the integrated intensity of the nucleus edge, and the contrast of the nucleus texture (Fig. 4E). In translation, tumor nuclei from high TiRS tiles exhibited significant larger size, more distorted shape, while their nuclear envelope and textural contrast were less prominent (Fig. 4C). Another set of measurements that reflected the heterogeneity of nuclear size and shape in one tile was also significantly correlated with high risk (Fig. 4E).

Together, these results showed that fine-grained features with prognostic significance were captured by prognostic model 2.

### The prognostic model reflects tumor biological processes at multi-omics scale

**Fig. 5:**
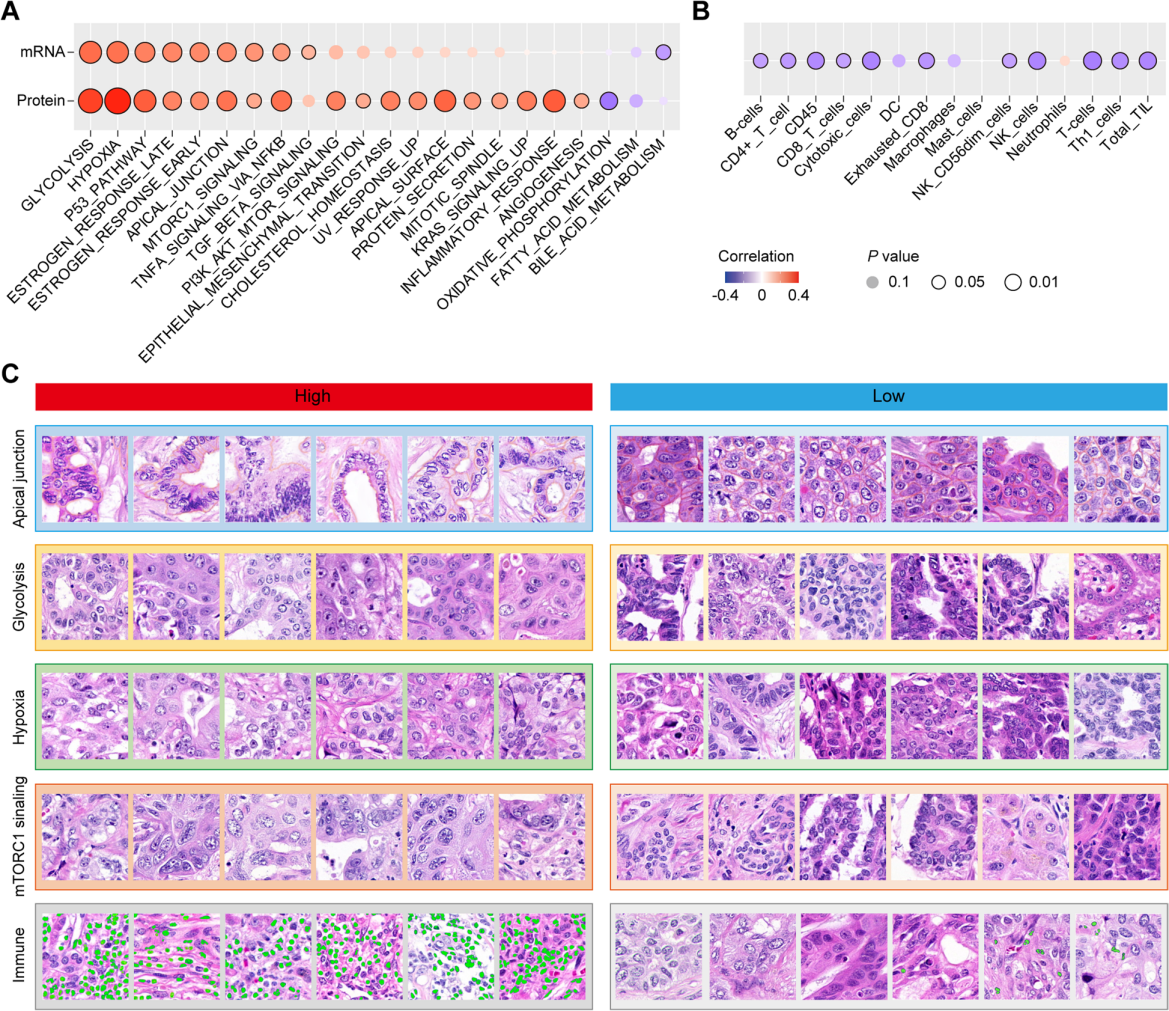
Relevance of TiRS to molecular alterations. **A** Correlation of TiRS with hallmark gene sets of cancer. The upper panel was based on transcriptomics data, and the lower panel was based on proteomics data. **B** Relevance of TiRS to tumor immune infiltration. **C** Comparison of tiles with opposing gene set scores that were morphologically discernible. Infiltrating immune cells were marked in green. TiRS, tile risk score

Logically, morphological patterns observable in histopathological sections and patient prognosis are determined by the underlying molecular phenotype [25, 26]. Leveraging the multi-omics data from FU-iCCA cohort [13], we identified several molecular alterations that were significantly associated with TiRS. The most relevant pathways revealed by transcriptomics data included glycolysis, hypoxia, P53 pathway, estrogen response, apical junction, mTORC1 signaling, TNFα signaling via NFKB, TGF-β signaling, and bile acid metabolism (Fig. 5A, Additional file 8: Fig. S6). Except for bile acid metabolism and fatty acid metabolism, all other pathways were positively correlated with TiRS, consistent with their adverse roles in cancer biology [27–32]. A similar correlation was also found between TiRS and proteomics data, which further confirmed the robust linkage between these hallmark molecular alterations and tumor cell morphology (Fig. 5A, Additional file 8: Fig. S6A).

We also analyzed the relevance of TiRS to immune cells based on RNA-seq data. In comparing immune milieu between low- and high-risk patients, we found that most T cell subsets, B cells, and NK cells had a statistically significant decrease in patients with high TiRS, while neutrophils demonstrated an opposite trend (Fig. 5B). These results complied with the complex functionality of immune phenotypes in cancer biology [33] and revealed that immunological status was also attended by the network.

Several morphological cues were recognized through pathologists’ vision by comparing tiles from samples with top or bottom pathway scores. As shown in Fig. 5C, tiles with top apical junction scores exhibited typical polarized tumor cell alignments, in contrast to the disorganized cell arrangements in comparing tiles. Tumor cells in tiles tagged as glycolysis appeared higher levels of cell spreading, comparing to the stacking appearance of the counterpart tiles. We also noted the swelling of the tumor cell under hypoxia and the distortion of tumor nuclei with high mTORC1 signaling scores. The basis of these observable morphological changes could be traced to the impact of their underlying molecular alterations on cellular interaction, cytoskeleton contractility, membrane permeability, and aberrant biogenesis of nuclear matter [27, 34–36].

However, most recurrent genomic alterations (such as *TP53*, *FGFR2*, and *IDH1/2*) did not correlate with TiRS, except for *KRAS* mutation (Additional file 8: Fig. S6B), which implied that most single mutations might not leave a strong enough footprint in morphology.

## Discussion

Despite recent progress, current deep learning-based prognostic models still struggled with poor consistency and generalizability [1]. Under canonical framework, it is almost impossible to discern if the model has attended the intrinsic prognostic features from histology or been misguided by confounders due to limitation and lopsidedness of the training datasets. In this study, we focused on interpretability of the deep learning model for iCCA, to explore the most relevant features that contribute to patient prognosis. To make the network more “transparent,” we first adopted a stepwise modeling strategy, by incorporating features derived from tissue- or cell-level dimensions into separate preliminary models. Then, we conducted extensive interrogative approaches to the final model, and potential prognostic features that were attended by the neural networks were extracted from the tremendous image information.

The architectural features that reflected the geographical organization and spatial interaction of essential tissue components were the determining parameters of prognostic model 1. We found that the distribution of TLSs provided a major cue for the prognostic prediction by this model. The distinct functional orientations and cellular compositions of TLSs located within or around the tumor were recently reported by our group, where intra-tumor TLSs suggested favorable prognosis while their peri-tumor counterparts were associated with dismal outcomes [12]. However, without this prior knowledge, the neural network could spontaneously comprehend the complex role of this immune feature. Another prominent feature was the protrusions and depressions along the invasive margin, which conformed to the presence of tumor budding (Additional file 6: Fig. S4). Tumor budding is an emerging prognostic biomarker that defined as cancer cell clusters protruded at the invasive front, suggesting the invasiveness and epithelial-mesenchymal transition of the tumor [20]. Despite that the prognostic value of tumor budding is well established in colorectal cancer [37], its potential application in iCCA warrants further investigation. Other visualized prognostic features, including tumor parenchyma/stroma ratio, disseminated foci, and tumor-adjacent micro-vessels, were all comprehensible to pathologists [21–23], yet the translation of these features into quantified risk scores was impossible by conventional pathological procedures.

Cell-level information was captured by prognostic model 2 from sampled tiles. We identified tumor parenchyma to be the most informative region, in line with a prior study [6]. Despite the known role of non-tumor cells in tumor microenvironment [21], our results and others [6, 12] implied that the functional states of them might be reflected by their distribution or density rather than cell morphology (such as TLS) and thus could not be attended by prognostic model 2. Tumor cell morphology, on the other hand, contained abundant biological imprints that could be perceived by both pathologists and neural networks. Tumor nuclei from high-risk tiles tended to exhibit increased size, more distorted shapes, and anomalous textures. Ploidy as an important determinant of nuclear size might be a plausible mechanism underlying the nuclear enlargement in iCCA tiles with high TiRS [38], given that aggressive iCCAs tend to display higher aneuploidy [39]. Likewise, the association of nuclear shape irregularities and specific molecular alterations that contributed to increased malignancy have been established in some other cancers, such as papillary thyroid carcinoma with *RET* fusion [40] and *EGFR* mutated lung adenocarcinomas [41]. The textural features of nuclei were mostly determined by chromatin pattern, with resultant changes in chromatin stability and gene expression states [42]. Heterochromatin, which is densely packed, transcriptionally silent, and located at the nuclear periphery, might explain the prominent nuclear envelope and texture in tiles with low TiRS. In contrast, nuclei in tiles with high TiRS exhibited more dispersed textures, which morphologically and functionally complied with the transcriptionally active euchromatin [42].

We also demonstrated that the neural network captured the footprints left by major molecular alterations that correlated with outcome. The significant gene sets that positively associated with TiRS reflected the proliferation (mTORC1 signaling and P53 pathway) [27, 29], cell skeleton and junction (apical junction) [28], and metabolism (glycolysis and hypoxia) of tumor cells, while bile acid metabolism and immune infiltration (except neutrophils) were negatively associated. Interestingly, a previous reported iCCA molecular subtypes shared similar patterns with us [39]. Subtype C1 from this study was enriched with *P53* mutations, microtubule cytoskeleton, and increased aneuploidy, and associated with dismal outcome. In contrast, subtype C2 with significantly better survival was linked with bile acid metabolism and increased leukocyte infiltrates (except myeloid cells) [39]. Indeed, some of the molecular alterations left discernible imprints in morphology [43]. For instance, tumor cells characterized by glycolysis exhibited more rigid shapes and higher levels of cell spreading, coinciding with the mechanical regulation of glycolysis via cytoskeleton stress [35]. The swelling of the tumor cells under hypoxia might correlate with the resultant cell membrane permeability change [36]. The hyperactivation of mTORC1 pathway in tumor cells continuously promoted the synthesis of nucleotides and the biogenesis of ribosome, which might induce tumor nuclei irregularities [27].

Our deep learning-based prognostic model for iCCA was deconstructed at unprecedented resolution and detail, and most of the features were comprehensible to pathologists with prior knowledge. This interpretability attributed to the proactive exclusion of irrelevant artifacts by the confinement of model inputs, and the accurate perception of determining prognosticators by the attention mechanism. Our model would not pursue the highest accuracy in the training cohort but demonstrated excellent robustness and generalizability when applying to the validation cohorts without any tuning. Another advantage of our model is the concise framework leaning on the resources of a personal computer, making it promising for wide range application.

## Conclusions

In conclusion, we proposed an interpretable deep learning framework to gain insights into the biological behavior and clinical outcome of iCCA. The final predictive model provides a comprehensive histopathological representation by extracting topological and fine-grained information simultaneously. We have demonstrated that most of the significant prognosticators perceived by the networks are comprehensible to human minds, thus helping to build up the trust needed to convince clinical users to rely on these computer-aided devices.

### Supplementary Information

Additional file 1: Supplementary methods

Additional file 2: Fig. S1. The study population and frameworks of the networks.

Additional file 3: Fig. S2. Typical examples of misclassified tiles.

Additional file 4: Fig. S3. C-indices of different parameters and subgroup analysis.

Additional file 5: Table S1. Univariate and multivariate analysis of overall survival.

Additional file 6: Fig. S4. Illustration of tumor budding.

Additional file 7: Fig. S5. Comparison of C-indices for architectural parameters.

Additional file 8: Fig. S6. Relevance of TiRS to molecular alterations.

## Data Availability

The data that support the findings of this study are available from the corresponding author upon reasonable request.

## Abbreviations

AUC: Area under the curve
C-index: Harrell’s concordance index
CRS: Consensus risk score
GS: GSM score
GSM: Global segmentation map
HN: Hemorrhage and necrosis region
iCCA: Intrahepatic cholangiocarcinoma
LT: Peri-tumor liver tissue
OSM: Occlusion sensitivity map
TiRS: Tile risk score
TLS: Tertiary lymphoid structure
TT: Tumor tissue
TT-p: Tumor parenchyma
TT-s: Tumor stroma
WSI: Whole slide image
