# Supplementary material for: Mining the interpretable prognostic features from pathological image of intrahepatic cholangiocarcinoma using multi-modal deep learning
Source: BMC Med. 2024 Jul 8;22:282. doi: 10.1186/s12916-024-03482-0 (PMC11229270; doi:10.1186/s12916-024-03482-0)
Supplement: Supplementary file 1 — Additional file 1: Supplementary methods [file 12916_2024_3482_MOESM1_ESM.docx]

**Additional file 1: Supplementary methods**

**1.1 Detailed information of the WSIs**

All WSIs were scanned using a fully automated digital pathology slide system (KF-PRO-120 from KFBIO Co., Ltd.) in KFB format. To read slides in Cohorts T, V1 and V2 when coding with Python, we converted the KFB files to the SVS format. The resolution range of the 89 WSIs that were randomly selected for manual annotation and the WSIs of all four cohorts are shown in the table below.

**The resolution range of the WSIs.**

| Cohort | Height | | | Width | | |
| --- | --- | --- | --- | --- | --- | --- |
|  | Min | Max | Average | Min | Max | Average |
| 89 WSIs for annotation | 50176 | 87040 | 76235.03 | 66000 | 184000 | 121494.25 |
| T | 26624 | 89088 | 76307.71 | 36000 | 186000 | 116182.72 |
| V1 | 39733 | 88064 | 79530.54 | 56061 | 184000 | 147935.34 |
| V2 | 46080 | 89088 | 70509.71 | 52000 | 126000 | 78976.19 |
| FU-iCCA | 182016 | 201216 | 200259.98 | 78848 | 86784 | 86388.85 |

Abbreviations: WSI, whole slide image

**1.2 Detailed framework for the classification networks**

We redesigned the ResNet18 model to make it suitable for tissue classification in iCCA. Specifically, we added two fully connected layers after the pooling layer as two branches correspondingly for TT/LT and TLS/HN classification. Based on the TT/LT prediction, we established a ResNet18 model for TT-p/TT-s classification.

When distinguishing the TT from LT or distinguishing the TT-p and TT-s regions, we added a fully connected layer containing one neuron as the output based on the redesigned model to classify the current input image block. Since the activation function used was the sigmoid function, the prediction result depends on the predicted probability. Similarly, when distinguishing the TLSs, and HN regions in WSIs, a fully connected layer containing 3 neurons was added as an output based on the redesigned model. The output result with high confidence was regarded as the final classification result.

The loss function used to optimize the classification networks was the cross-entropy function, which was defined as follows.

$$L_{cls}= \frac{1}{N}\sum_{i} -[y_{i}*\log(p_{i}) + (1-y_{i})*\log(1-p_{i})]$$

where $y_{i}$ denotes the true category of the $i^{th}$ tissue block and $p_{i}$ is the prediction probability by model.

As demonstrated in Figure S1, WSIs were cropped into non-overlapping $256\times256$ pixels tiles. The cropped tiles were fed to the classification model built for TT/LT and TLS/HN identification. Then TT-p/TT-s prediction was assigned to tiles recognized as TT through a binary classification ResNet18.

**1.3 Details of the prognostic networks and the Cox regression model**

WSIs from Zhongshan Hospital of Fudan University were randomly divided into the training set (Cohort T, 673 WSIs, 373 patients) and the validation set (Cohort V1, 433 WSIs, 213 patients).

As shown in Figure S1, ResNet50 and ResNet18 were employed correspondingly for prognostic model 1 (GSM-based) and prognostic model 2 (tile-based) for risk prediction. The extracted features after average pooling were fed to a fully connected layer with the linear activation to predict the hazard rate (risk) of samples. To integrate these two models, we concatenated their outputs as a two-dimensional vector and created a fully connected layer to predict the risk score from the concatenated vector. Therefore, the fully connected layer in the integrated model took a two-dimensional vector as input and outputs a single scalar with a linear activation function. We used weights from trained tile-based and GSM-based preliminary prognostic models to initialize weights in the integrated model. Then, the integrated model was trained in an end-to-end manner.

For optimization, the loss function aimed at maximizing log likelihood following Cox. Let $h_{i}$ denote the predicted hazard rate of a sample $x_{i}$, the loss function is defined as:

$$L=-\sum_{x_{i}\in\mathbb{D}_{ucs}} (h_{i}-log\sum_{j:t_{j}\geq t_{i}} exp(h_{i}))$$

where $\mathbb{D}_{ucs}$ denotes the set of uncensored data and $t_{i}$ denotes the true survival time of $x_{i}$.

For all employed models, we used the Adam optimizer during training. For the prognostic model 1, we used the learning rate of 0.001 and the weight of decay 0.0001. For the prognostic model 2, we used the learning rate 0.01 and the weight of decay 0.0001. Before training the integrated model, we initialized the weights in the integrated model except for the final fully-connected layer with the optimized weights from the prognostic models 1 and 2. Then, the integrated model was optimized with the learning rate 0.0001 and the weight of decay 0.0001.

We also tested the predictive performance of the direct combination of GS and TiRS by using hazard linear combination. Specifically, we aggregated GS and TiRS by linear combination with learnable weights for hazard regression, namely hazard linear combination.

For performance comparison, we established a Cox regression model based on the clinical and pathological variables of Cohort T. The following factors were found to be independent predictors: tumor size, tumor number, microvascular invasion (MVI), lymph node metastasis (LNM) and CA19-9. Then the Clinical index could be calculated based on the equation obtained from multivariate Cox regression:

Clinical index = (0.503 * Tumor size) + (0.686 * Tumor number) + (0.505 * MVI) + (0.857 * LNM) + (0.411 * CA19-9)

**1.4 Detailed methodology for Occlusion Sensitivity Map (OSM) and predefined architectural parameters**

To spatially evaluate the effect of segmentation on risk prediction, the GSM was occluded through setting a subregion of $30\times30$ pixels as background, and the corresponding predicted hazard rate was compared with the hazard rate of the original GSM. Therefore, the influence of one spatial position was evaluated through the difference between hazard rates of occluded and original GSM. For further understanding and visualization, we defined the OSM as a map of hazard rate difference generated with a sliding window of $30\times30$ pixels and the stride of 4.

We also defined some architectural parameters based on predicted GSM. Such predefined architectural parameters included three types: area ratio, distance and smoothness. The area ratio was based on the number of pixels in a distinct region. The distance between two regions was defined as Euclidean distance between mean coordinates of points in these two regions. For smoothness estimation, we employed Sobel operator to evaluate spatial differences on the boundary of one region. Since defined gradients and distances were sequences with variable lengths, mean and variance were used to describe the distribution of these two parameters.

**1.5 Detailed description of CellProfiler pipeline**

For tile-level analysis, we extracted features from tiles using CellProfiler. In this subsection, the detailed pipeline of feature extraction is demonstrated.

First, the RGB tile was unmixed into hematoxylin and eosin channels. With the hematoxylin channel, objects of nucleus were identified. Based on identified nucleus objects, features of texture, intensity and shape were extracted from corresponding object areas. Mean, median, standard deviation values of such features were used for image-level description.
