# Supplementary material for: Mining the interpretable prognostic features from pathological image of intrahepatic cholangiocarcinoma using multi-modal deep learning
Source: BMC Med. 2024 Jul 8;22:282. doi: 10.1186/s12916-024-03482-0 (PMC11229270; doi:10.1186/s12916-024-03482-0)
Supplement: Supplementary file 2 — Additional file 2: Fig. S1. The study population and frameworks of the networks. [file 12916_2024_3482_MOESM2_ESM.docx]

**Additional file 2: Fig. S1**

**
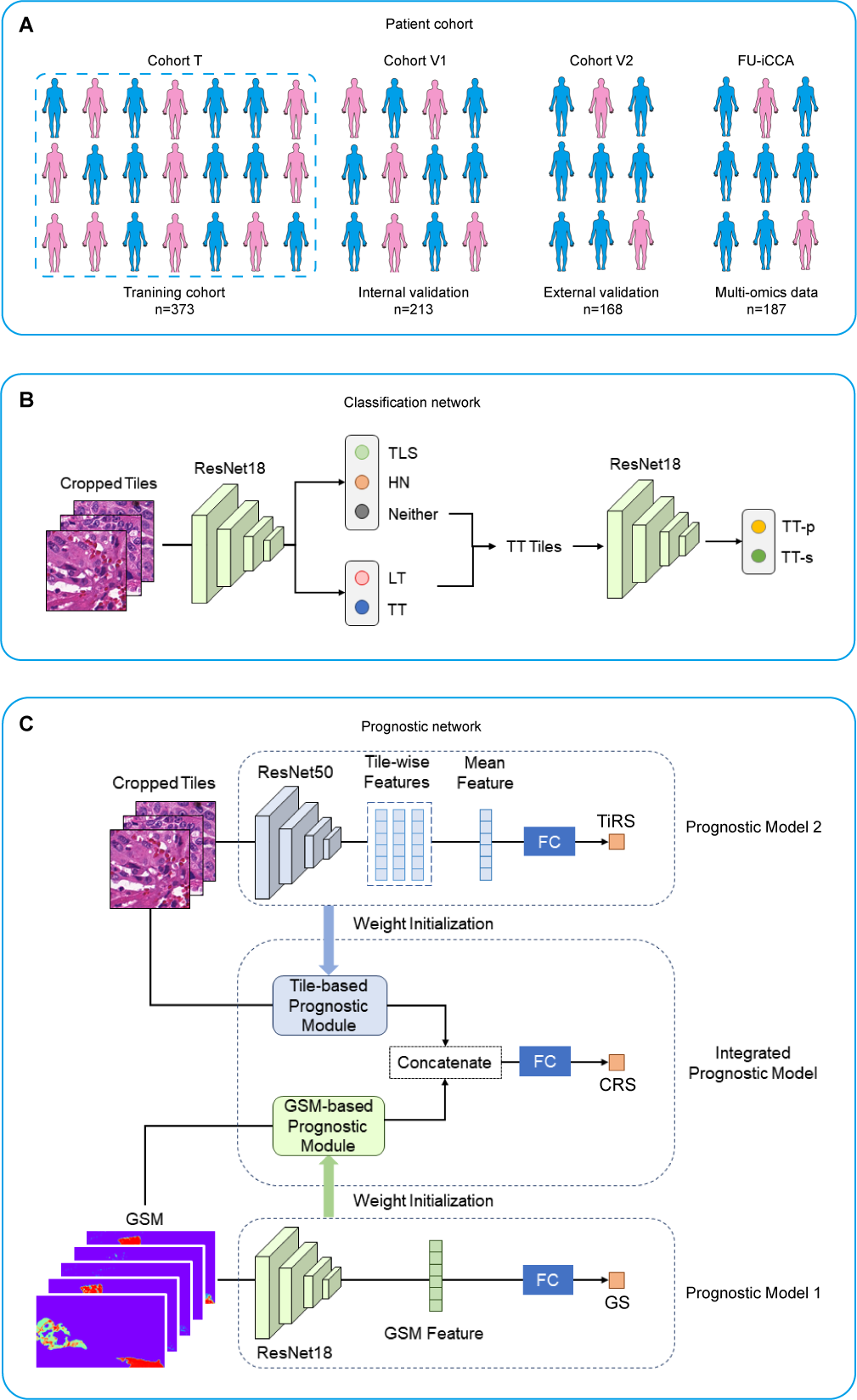
**

**Figure S1.** (A) The study population and patient cohorts. (B) The framework for the classification networks. (C) The framework for the prognostic networks. TLS: tertiary lymphoid structure; HN: hemorrhage and necrosis; LT: peri-tumor liver tissue; TT: tumor tissue; TT-p: tumor parenchyma; TT-s: tumor stroma. TiRS: tile risk score; CRS: consensus risk score; GSM: global segmentation map; GS: GSM score; FC: fully connected layer.
