# Supplementary material for: Mining the interpretable prognostic features from pathological image of intrahepatic cholangiocarcinoma using multi-modal deep learning
Source: BMC Med. 2024 Jul 8;22:282. doi: 10.1186/s12916-024-03482-0 (PMC11229270; doi:10.1186/s12916-024-03482-0)
Supplement: Supplementary file 3 — Additional file 3: Fig. S2. Typical examples of misclassified tiles. [file 12916_2024_3482_MOESM3_ESM.docx]

**Additional file 3: Fig. S2**


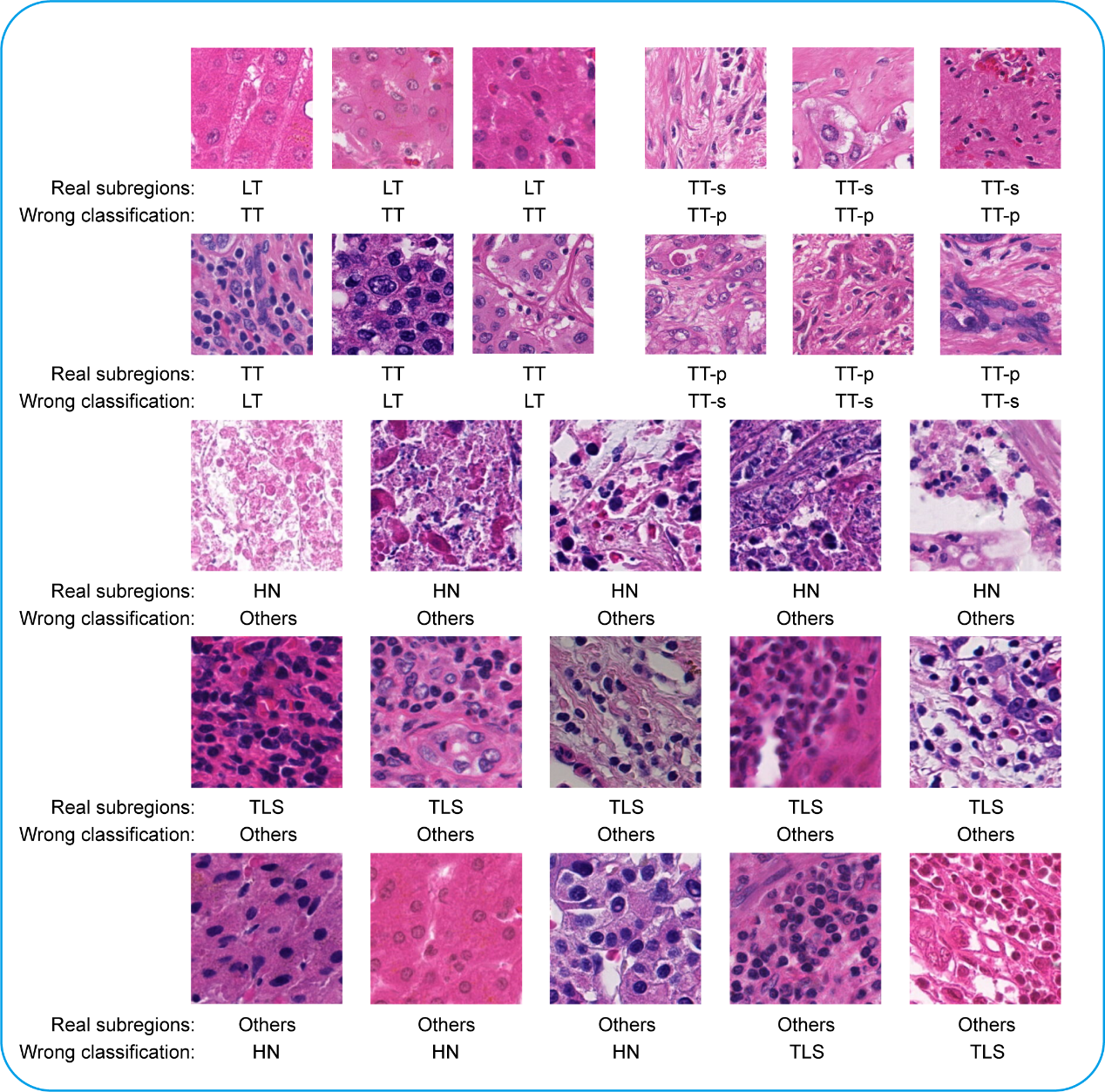


**Figure S2.** Typical examples of misclassified tiles. TT: tumor tissue; LT: peri-tumor liver tissue; TT-p: tumor parenchyma; TT-s: tumor stroma; HN: hemorrhage and necrosis; TLS: tertiary lymphoid structure.
