# Supplementary material for: Mining the interpretable prognostic features from pathological image of intrahepatic cholangiocarcinoma using multi-modal deep learning
Source: BMC Med. 2024 Jul 8;22:282. doi: 10.1186/s12916-024-03482-0 (PMC11229270; doi:10.1186/s12916-024-03482-0)
Supplement: Supplementary file 4 — Additional file 4: Fig. S3. C-indices of different parameters and subgroup analysis. [file 12916_2024_3482_MOESM4_ESM.docx]

**Additional file 4: Fig. S3**

**
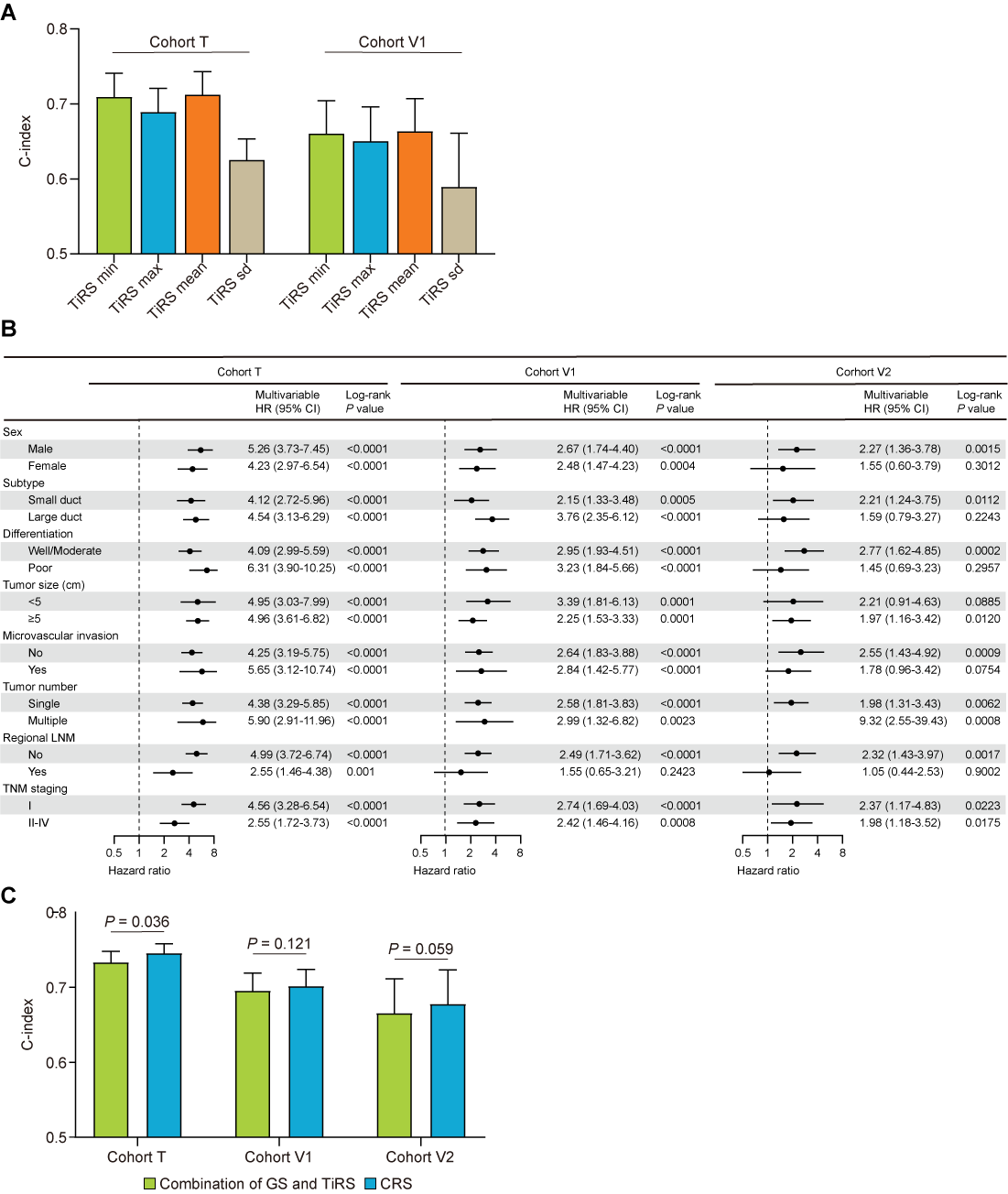
**

**Figure S3.** (A) C-indices via the minimal (TiRSmin), maximal (TiRSmax), mean (TiRSmean), and standard deviation (TiRSsd) of the TiRSs among patients with multiple WSIs in Cohorts T and V1. (B) Subgroup analysis and forest plots for overall survival illustrating multivariate hazard ratios according to CRS high versus low. (C) C-indices via the direct combination of GS and TiRS (using hazard linear combination) and CRS. The bars represent the 95% confidence intervals. TiRS: tile risk score; HR: hazard ratio; CI: confidence interval; LNM: lymph node metastasis; CRS: consensus risk score.
