# Supplementary material for: Mining the interpretable prognostic features from pathological image of intrahepatic cholangiocarcinoma using multi-modal deep learning
Source: BMC Med. 2024 Jul 8;22:282. doi: 10.1186/s12916-024-03482-0 (PMC11229270; doi:10.1186/s12916-024-03482-0)
Supplement: Supplementary file 5 — Additional file 5: Table S1. Univariate and multivariate analysis of overall survival. [file 12916_2024_3482_MOESM5_ESM.docx]

**Additional file 5: Table S1**

**Table S1: Univariate and multivariate analysis of overall survival in the three cohorts.**

|  | Cohort T (n=373) | | | |  | Cohort V1 (n=213) | | | |  | Cohort V2 (n=168) | | | |
| --- | --- | --- | --- | --- | --- | --- | --- | --- | --- | --- | --- | --- | --- | --- |
|  | Univariable | Multivariable | | |  | Univariable | Multivariable | | |  | Univariable | Multivariable | | |
| Variable | *P* value | HR | 95%CI | *P* value |  | *P* value | HR | 95%CI | *P* value |  | *P* value | HR | 95%CI | *P* value |
| Age, years (≤65 vs. >65) | 0.347 | - | - | - |  | 0.178 | - | - | - |  | 0.833 | - | - | - |
| Sex (female vs. male) | 0.472 | - | - | - |  | 0.836 | - | - | - |  | 0.137 | - | - | - |
| HBsAg (negative vs. positive) | 0.779 | - | - | - |  | 0.007 | 0.614 | 0.397-0.956 | 0.033 |  | 0.611 | - | - | - |
| CA19-9, U/ml (≤37 vs. >37) | <0.001 | 0.883 | 0.644-1.208 | 0.436 |  | <0.001 | 0.661 | 0.445-0.979 | 0.041 |  | 0.039 | 0.677 | 0.423-1.083 | 0.112 |
| Tumor number (solitary vs. multiple) | <0.001 | 0.465 | 0.322-0.673 | <0.001 |  | 0.207 | - | - | - |  | 0.344 | - | - | - |
| Tumor size, cm (≤5 vs. >5) | <0.001 | 0.735 | 0.547-0.989 | 0.042 |  | <0.001 | 0.559 | 0.372-0.839 | 0.006 |  | <0.001 | 0.547 | 0.329-0.915 | 0.021 |
| Differentiation (Well/Moderate vs. Poor) | 0.055 | 0.928 | 0.675-1.274 | 0.637 |  | 0.840 | - | - | - |  | 0.973 | - | - | - |
| Lymph node metastasis (no vs. yes) | <0.001 | 0.432 | 0.295-0.611 | <0.001 |  | <0.001 | 0.464 | 0.287-0.752 | 0.002 |  | 0.002 | 0.860 | 0.496-1.488 | 0.561 |
| Microvascular invasion (no vs. yes) | 0.022 | 0.856 | 0.576-1.269 | 0.436 |  | 0.002 | 0.625 | 0.395-0.987 | 0.045 |  | <0.001 | 0.361 | 0.223-0.592 | <0.001 |
| Macrovascular invasion (no vs. yes) | 0.001 | 0.566 | 0.338-0.942 | 0.028 |  | 0.029 | 0.782 | 0.396-1.542 | 0.478 |  | 0.326 | - | - | - |
| CRS (Low vs. High) | <0.001 | 0.264 | 0.186-0.387 | <0.001 |  | <0.001 | 0.409 | 0.267-0.623 | <0.001 |  | <0.001 | 0.419 | 0.222-0.732 | 0.002 |

Abbreviations: CI, confidence interval; HBsAg, hepatitis B surface antigen; CRS, consensus risk score.
