# Supplementary material for: Mining the interpretable prognostic features from pathological image of intrahepatic cholangiocarcinoma using multi-modal deep learning
Source: BMC Med. 2024 Jul 8;22:282. doi: 10.1186/s12916-024-03482-0 (PMC11229270; doi:10.1186/s12916-024-03482-0)
Supplement: Supplementary file 6 — Additional file 6: Fig. S4. Illustration of tumor budding. [file 12916_2024_3482_MOESM6_ESM.docx]

**Additional file 6: Fig. S4**


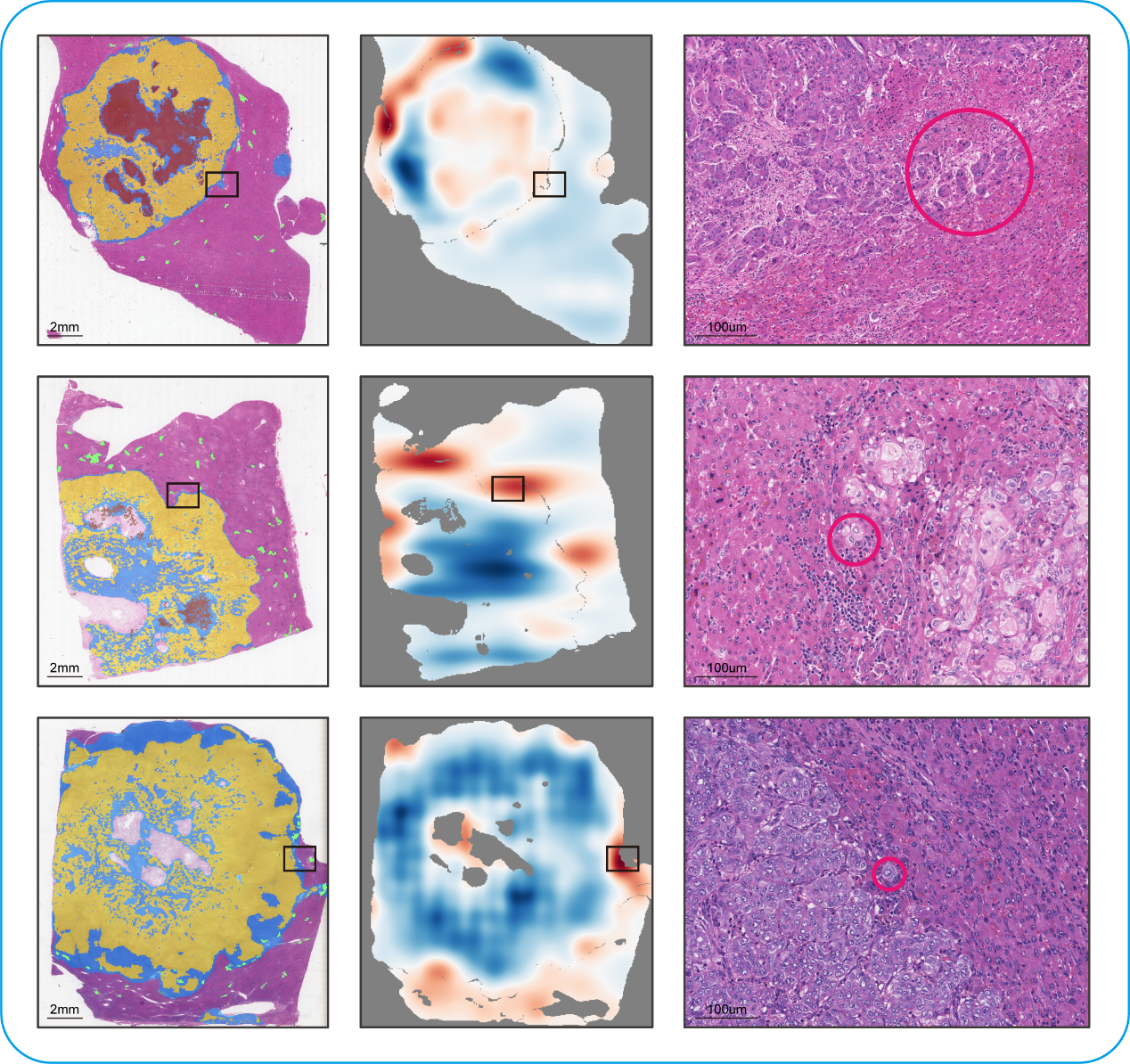


**Figure S4.** The presence of tumor budding at the protrusions and depressions along the invasive margin of intrahepatic cholangiocarcinoma. Tumor budding was indicated by red circle.
