# Supplementary material for: Mining the interpretable prognostic features from pathological image of intrahepatic cholangiocarcinoma using multi-modal deep learning
Source: BMC Med. 2024 Jul 8;22:282. doi: 10.1186/s12916-024-03482-0 (PMC11229270; doi:10.1186/s12916-024-03482-0)
Supplement: Supplementary file 7 — Additional file 7: Fig. S5. Comparison of C-indices for architectural parameters. [file 12916_2024_3482_MOESM7_ESM.docx]

**Additional file 7: Fig. S5**

**
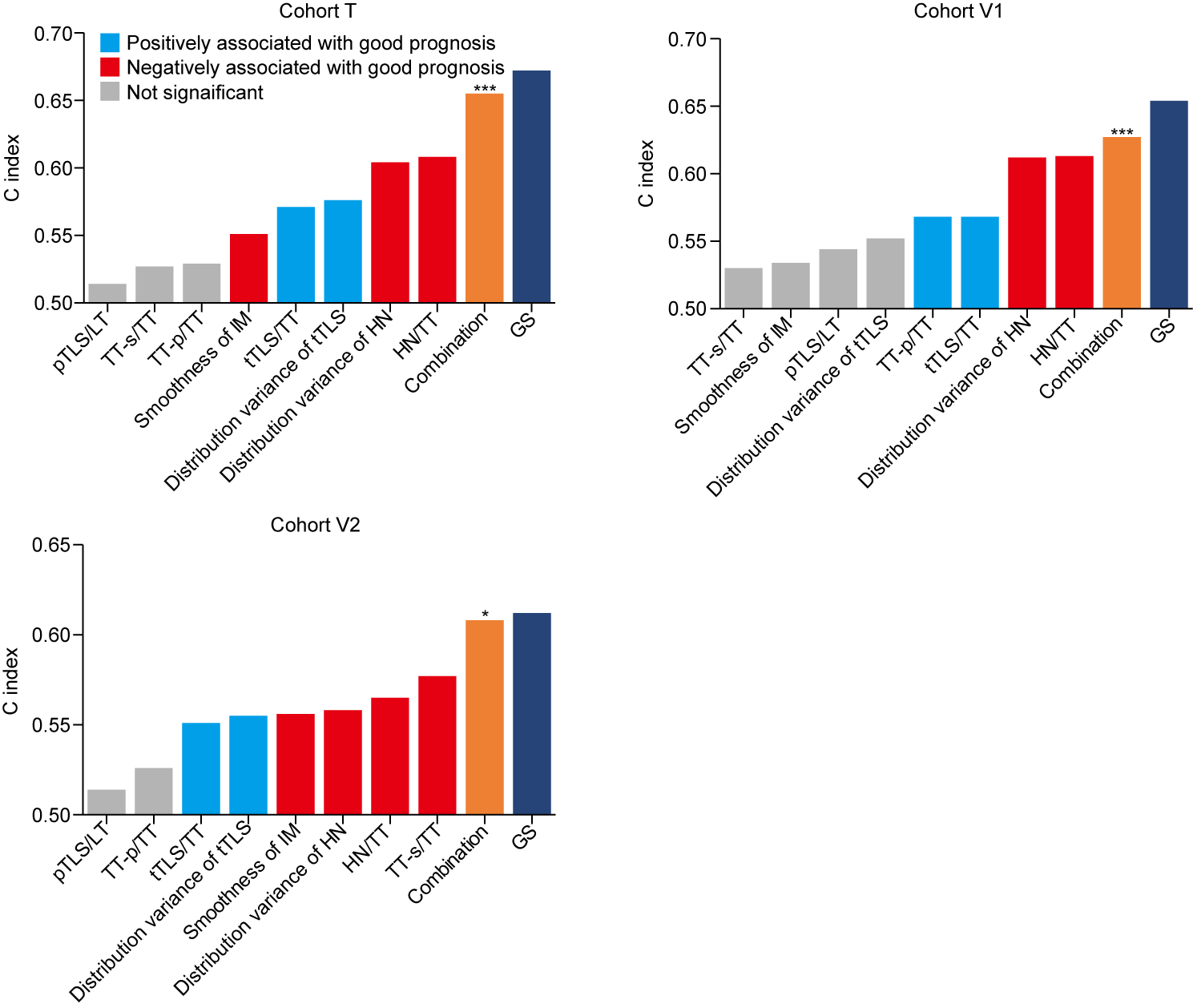
**

**Figure S5.** Comparison of C-indices for predefined architectural parameters, the combination of these parameters and GS. *, *P* <0.05; ***, *P* <0.001. TT, tumor tissue; LT, peri-tumor liver tissue; TLS, tertiary lymphoid structure; HN, hemorrhage and necrosis; IM: invasive margin; pTLS: peri-tumor TLS; tTLS: intra-tumor TLS; TT-s: tumor stroma; TT-p: tumor parenchyma; GS: GSM score; GSM, global segmentation map.
