# Supplementary material for: Mining the interpretable prognostic features from pathological image of intrahepatic cholangiocarcinoma using multi-modal deep learning
Source: BMC Med. 2024 Jul 8;22:282. doi: 10.1186/s12916-024-03482-0 (PMC11229270; doi:10.1186/s12916-024-03482-0)
Supplement: Supplementary file 8 — Additional file 8: Fig. S6. Relevance of TiRS to molecular alterations. [file 12916_2024_3482_MOESM8_ESM.docx]

**Additional file 8: Fig. S6**


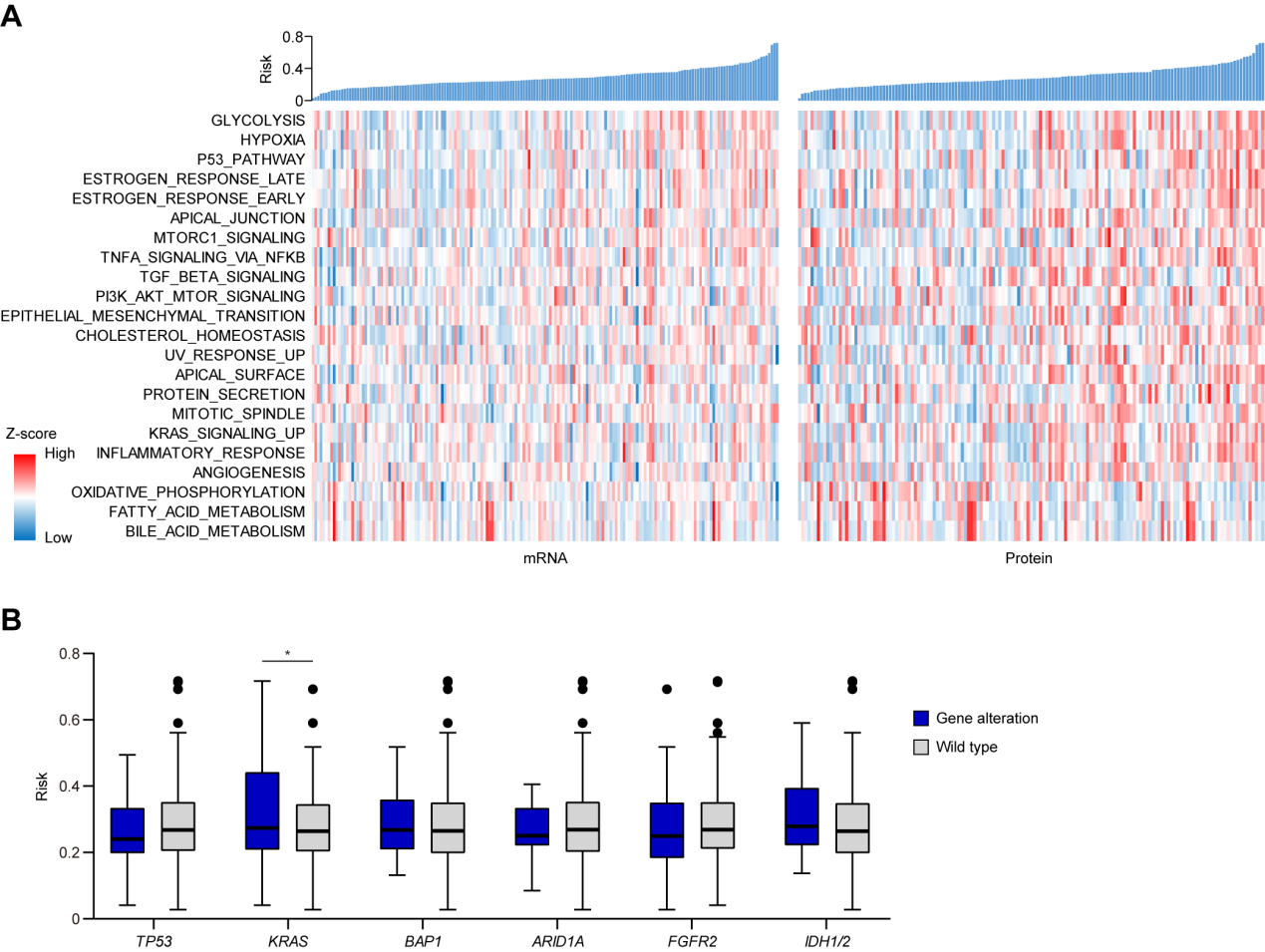


**Figure S6.** (A) Heatmaps shows the relevance of TiRS to hallmark gene sets of cancer. (B) Relevance of TiRS to recurrent genomic alterations. Boxes represent 25%-75% percentiles; range bars represent the 5% and 95% percentiles. *: *P* <0.05
